# Prevalence of Vitamin B12 Deficiency Among Patients With Type 2 Diabetes Mellitus on Long-Term Metformin Therapy

**DOI:** 10.7759/cureus.103178

**Published:** 2026-02-07

**Authors:** Vikas Rangare, Jyoti Nagwanshi, Kapil Raghuwanshi

**Affiliations:** 1 Department of Internal Medicine, Chhindwara Institute of Medical Sciences, Chhindwara, IND; 2 Department of Medicine, Chhindwara Institute of Medical Sciences, Chhindwara, IND; 3 Department of Biochemistry, Chhindwara Institute of Medical Sciences, Chhindwara, IND

**Keywords:** diabetes complications, long-term metformin therapy, metformin, neuropathy, prevalence, proton pump inhibitors, type 2 diabetes mellitus, vitamin b12 deficiency

## Abstract

Background: Vitamin B12 insufficiency, which aggravates hematological and neurological problems in diabetics, has been linked to chronic metformin therapy, the main treatment for type 2 diabetes mellitus (T2DM). This study aimed to assess the prevalence of vitamin B12 deficiency in patients with T2DM on metformin and identify associated clinical and demographic factors in an Indian tertiary care setting.

Methodology: An observational cross-sectional hospital-based study was conducted among adults with T2DM. Sociodemographic data, clinical history, diabetes duration, medication use, and laboratory parameters were recorded. Serum vitamin B12 levels were measured using standard laboratory cutoffs to identify deficiency, and data were analyzed using appropriate descriptive and inferential statistical methods.

Results: Among 170 participants, multivariate logistic regression analysis identified metformin use for more than five years (adjusted odds ratio (OR): 2.85; 95% confidence interval (CI): 1.32-6.12), concomitant pantoprazole use (adjusted OR: 2.41; 95% CI: 1.08-5.34), and low high-density lipoprotein cholesterol levels below 40 mg/dL (adjusted OR: 2.22; 95% CI: 1.01-4.91) as independent predictors of vitamin B12 deficiency. Low hemoglobin levels, elevated low-density lipoprotein cholesterol levels, and poor glycemic control were not significantly associated (p > 0.05).

Conclusion: Patients with T2DM, especially those on long-term metformin medication, frequently lack vitamin B12. Timely supplementation and routine screening might help lower avoidable consequences.

## Introduction

Type 2 diabetes mellitus (T2DM) represents a widespread chronic metabolic disorder, contributing to a substantial and increasing global health burden. Current estimates indicate that over half a billion adults are affected by diabetes, with projections suggesting a significant rise in the coming years. This trend is particularly pronounced in South Asian countries such as India, where genetic predisposition, evolving lifestyles, and dietary patterns are key contributors to the epidemic. Consequently, effective management of T2DM is crucial to mitigate long-term vascular complications, which are primary drivers of diabetes-related morbidity and mortality [1,2].

Metformin remains the cornerstone of pharmacological management for T2DM owing to its efficacy, safety, and affordability. However, evidence from over the years indicates that chronic metformin therapy impairs vitamin B12 absorption, thereby increasing the risk of deficiency. Given that neurological manifestations of vitamin B12 deficiency may be misattributed to diabetic complications, unrecognized deficiency can result in delayed diagnosis and management [3,4].

Undiagnosed vitamin B12 deficiency in patients with T2DM can have serious clinical consequences. It may lead to hematological abnormalities such as megaloblastic anemia and neurological manifestations, including peripheral neuropathy, cognitive impairment, and autonomic dysfunction. Given the high prevalence of diabetic neuropathy in this population, a concurrent vitamin B12 deficiency can either mask or exacerbate neurological symptoms, potentially resulting in delayed diagnosis and suboptimal management. Additionally, high homocysteine levels are linked to vitamin B12 deficiency, which may raise cardiovascular risk and exacerbate diabetic patients' pre-existing vascular susceptibility [5,6].

Several concomitant conditions may increase the risk of vitamin B12 deficiency in the diabetic population. Prolonged use of acid-suppressing drugs, such as proton pump inhibitors (PPIs), which are frequently used for gastroesophageal disorders, lowers the production of gastric acid. This reduction can impair the separation of vitamin B12 from dietary proteins, a necessary step for its absorption. Additionally, underlying metabolic disturbances frequently observed in diabetes, such as chronic hyperglycemia, dyslipidemia, and declining renal function, may further disrupt the normal metabolism and utilization of vitamin B12. However, the precise nature of these relationships remains to be investigated [7,8].

Despite its clinical significance, vitamin B12 deficiency remains under-recognized and underdiagnosed in diabetic populations, particularly in India. Contributing factors include limited awareness among healthcare providers, overlapping clinical features with diabetic complications, and a lack of routine screening protocols. Assessing the prevalence of vitamin B12 deficiency in this population is therefore essential for developing effective preventive strategies, such as targeted screening and supplementation for high-risk individuals [9,10].

The current study also assessed the impact of concurrent PPI therapy on vitamin B12 status because, in addition to metformin exposure, patients with T2DM frequently use acid-suppressive therapy, such as PPIs, which may further impair vitamin B12 absorption by reducing gastric acid secretion.

There is still little data from local populations, despite mounting evidence from around the world. Understanding the severity of vitamin B12 deficiency and the factors associated with it in individuals with T2DM is necessary for making informed clinical decisions. To ascertain the prevalence of vitamin B12 deficiency among patients with T2DM and to evaluate its correlation with specific clinical and demographic characteristics, the current investigation was conducted.

## Materials and methods

After receiving approval from the Institutional Human Ethics Committee of Chhindwara Institute of Medical Sciences (approval number: CIMS/Ethics Committee/2024/8514), this hospital-based observational cross-sectional study was carried out at District Hospital Chhindwara, associated with Chhindwara Institute of Medical Sciences, Chhindwara, Madhya Pradesh, India, over a one-year period from January 2024 to December 2025.

Study population and sampling

Because metformin interferes with vitamin B12 absorption in the distal ileum and a chronic shortage may affect DNA synthesis in red blood cell precursors, resulting in megaloblastic anemia, serum vitamin B12 levels were measured. Since acid suppression can further decrease the absorption of vitamin B12, concurrent usage of PPIs was also seen. Multivariate analysis was employed to find independent predictors of deficiency, and comparisons between high and low dose and duration groups were carried out.

Throughout the study period, adult patients (≥18 years old) who visited the Department of Medicine's inpatient and outpatient departments were successively screened for eligibility. The study comprised patients who had been diagnosed with T2DM according to the American Diabetes Association's (ADA) criteria [11] and who had been taking metformin continuously for at least a year. Until the necessary sample size was reached, consecutive sampling was used. Written informed permission was obtained before enrolling eligible patients.

Individuals with T2DM or gestational diabetes, known chronic kidney disease, chronic liver disease, thyroid disorders, pregnancy or lactation, a history of vitamin B12 deficiency, current or recent (within the last six months) vitamin B12 supplementation, or use of drugs known to impede the absorption of vitamin B12 were excluded.

The eligibility of 236 patients was evaluated. Of these, 66 patients were excluded: 42 did not fit the inclusion criteria because they had been on metformin therapy for less than a year, had been diagnosed with type 1 or gestational diabetes mellitus, had chronic kidney or liver disease, or had thyroid disorders; 14 were taking vitamin B12 supplements or medications that were known to interfere with the absorption of vitamin B12; and 10 declined to participate. After giving their informed consent, the remaining 170 eligible patients joined the trial. As this was a cross-sectional study, there was no follow-up period, and all enrolled participants (170) were included in the final analysis.

Sample size calculation

The formula for estimating a single proportion was used to get the sample size: $\mathrm{n}=\mathrm{Z}^{2}\mathrm{p}\left(1&minus;\mathrm{p}\right)/\mathrm{d}^{2}$. Here, Z = 1.96 (95% confidence level), p = 0.30 (expected prevalence of vitamin B12 deficiency among metformin users based on previous studies), and d = 0.07 (absolute precision).

Based on the estimated 30% prevalence of vitamin B12 insufficiency among T2DM patients on long-term metformin therapy, the sample size was determined [12,13]. The minimum necessary sample size was 165 using a 95% confidence interval (CI) and 7% absolute precision; this was raised to 170 to allow for potential attrition. 

Data collection

Trained investigators conducted structured in-person interviews and clinical evaluations utilizing a pre-tested, standardized data collection form. Sociodemographic details (age, sex), the length of diabetes, the duration and daily dosage of metformin therapy, and concurrent pantoprazole use were all included in the data collection form. In order to reduce recollection bias, information about the use of medications (metformin and pantoprazole) was gathered from patient interviews and confirmed by looking through prescriptions and medical records when possible.

Based on previously published risk thresholds for vitamin B12 deficiency, the duration of metformin medication was measured in years and classified as ≤5 years and >5 years. In order to evaluate dose-dependent risk, the daily dose of metformin was recorded from medical records and assessed using a cutoff value of ≤2000 mg/day and >2000 mg/day.

Laboratory investigations

Following an overnight fast of at least 8-10 hours, each subject was evaluated in a laboratory. In accordance with internal quality control processes and standard operating procedures, venous blood samples were obtained and examined in the hospital's central clinical laboratory. Fasting blood glucose, postprandial blood glucose, glycated hemoglobin (HbA1c), hemoglobin levels, renal function tests (creatinine and serum urea), and a lipid profile that included triglycerides, low-density lipoprotein (LDL) cholesterol, and high-density lipoprotein (HDL) cholesterol were all examined in the lab. A standardized automated immunoassay technique (chemiluminescence-based test) was used to detect the levels of serum vitamin B12. The levels of serum vitamin B12 were classified as normal (>300 pg/mL), borderline (200-300 pg/mL), or deficient (<200 pg/mL).

Statistical analysis

The IBM SPSS Statistics for Windows, V. 23.0 (IBM Corp., Armonk, NY, USA), was used to evaluate the data after it was entered into Microsoft Excel (Microsoft Corp., Redmond, WA, USA). While categorical variables were summed up as frequencies and percentages, continuous variables were checked for normality and expressed as mean ± standard deviation. When comparing groups, the chi-squared test was used for categorical data and Student's t-test or the one-way analysis of variance (ANOVA) for continuous variables, if applicable. To find independent predictors of vitamin B12 deficiency, multivariate logistic regression analysis was used. Variables having a p-value of <0.20 on univariate analysis were added to the regression model. The Hosmer-Lemeshow goodness-of-fit test was used to evaluate the model's fit. P-values less than 0.05 were regarded as statistically significant. Complete-case analysis was used to manage the few missing data.

## Results

A total of 170 participants (90 males, 80 females) with a mean age of 55.4 ± 9.8 years were included, with no significant age difference between genders (p = 0.32). The mean body mass index (BMI) was 26.8 ± 3.9 kg/m², the mean duration of diabetes was 8.1 ± 4.3 years, and the mean duration of metformin therapy was 6.5 ± 3.1 years, all comparable between males and females (p > 0.05). The overall mean HbA1c was 7.8 ± 1.2, with no significant gender-based difference (p = 0.29) (Table 1).

**Table 1:** Baseline characteristics of the study participants Comparisons between males and females were performed using Student's independent t-test. Values are presented as mean ± standard deviation. A p-value of <0.05 was considered statistically significant. BMI: body mass index; HbA1c: glycated hemoglobin

Variable	Total (n = 170)	Male (n = 90)	Female (n = 80)	Statistical value	P-value
Age (years)	55.4 ± 9.8	56.2 ± 9.5	54.6 ± 10.1	0.99	0.32
BMI (kg/m²)	26.8 ± 3.9	26.5 ± 3.7	27.1 ± 4.2	-0.83	0.41
Duration of diabetes (years)	8.1 ± 4.3	8.3 ± 4.4	7.9 ± 4.2	0.57	0.57
Duration of metformin use (years)	6.5 ± 3.1	6.7 ± 3.2	6.3 ± 3.0	0.74	0.46
Mean HbA1c (%)	7.8 ± 1.2	7.9 ± 1.3	7.7 ± 1.1	1.07	0.29

Vitamin B12 deficiency (<200 pg/mL) was identified in 56 (32.9%) participants. Borderline vitamin B12 levels (200-300 pg/mL) were observed in 48 (28.2%) participants, while 66 (38.9%) participants had normal vitamin B12 levels (>300 pg/mL) (Table 2).

**Table 2:** Distribution of vitamin B12 levels

Vitamin B12 status	Total number (n)	Percentage (%)
Deficient (<200 pg/mL)	56	32.9
Borderline (200-300 pg/mL)	48	28.2
Normal (>300 pg/mL)	66	38.9
Total	170	100

Vitamin B12 deficiency was substantially correlated with both longer term (>5 years) and higher daily dose (>2000 mg/day) of metformin. The vitamin B12-deficient and vitamin B12-non-deficient groups were comparable in age (55.9 ± 9.5 vs. 55.2 ± 10.1 years), gender distribution, and mean HbA1c levels (7.9 ± 1.2 vs. 7.7 ± 1.3). In contrast, deficiency was more common in patients using metformin for more than five years, 40 (71.4%) vs. 53 (46.5%), and in those receiving daily doses >2000 mg, 36 (64.3%) vs. 44 (38.6%), indicating a significant association with longer duration and higher dosage of metformin therapy (Table 3).

**Table 3:** Association between vitamin B12 deficiency and clinical factors ^c^Chi-squared test. ^t^Student's t-test. *Significant p-value. Continuous variables are expressed as mean ± standard deviation and were compared using the independent (unpaired), two-tailed Student's t-test. Categorical variables are presented as numbers (percentages) and were compared using the chi-squared test. A p-value of <0.05 was considered statistically significant. HbA1c: glycated hemoglobin

Variable	Deficient (n = 56)	Non-deficient (n = 114)	Test values	P-value
Age (years, mean ± SD)	55.9 ± 9.5	55.2 ± 10.1	0.37^t^	0.71
Male sex	29 (51.8%)	61 (53.5%)	0.05^c^	0.83
Duration of metformin use >5 years	40 (71.4%)	53 (46.5%)	6.44^c^	0.01*
Metformin dose >2000 mg/day	36 (64.3%)	44 (38.6%)	8.20^c^	0.004*
HbA1c (%)	7.9 ± 1.2	7.7 ± 1.3	1.08^t^	0.28

Multivariate logistic regression analysis showed that metformin use for more than five years (adjusted odds ratio (OR): 2.85; 95% CI: 1.32-6.12; p = 0.007), concomitant pantoprazole use (adjusted OR: 2.41; 95% CI: 1.08-5.34; p = 0.031), and low HDL cholesterol <40 mg/dL (adjusted OR: 2.22; 95% CI: 1.01-4.91; p = 0.048) were independent predictors of vitamin B12 deficiency. Low hemoglobin (<12 g/dL), elevated LDL cholesterol (>130 mg/dL), and poor glycemic control (HbA1c >8%) were not significantly associated as the observed p-value was less than 0.05 (Table 4).

**Table 4:** Logistic regression predicting vitamin B12 deficiency *A p-value of <0.05 is considered statistically significant. OR: odds ratio; CI: confidence interval; LDL: low-density lipoprotein; HDL: high-density lipoprotein; Hb: hemoglobin

Predictor variable	Adjusted OR (95% CI)	P-value
Duration of metformin (>5 years)	2.85 (1.32-6.12)	0.007*
Pantoprazole use (yes)	2.41 (1.08-5.34)	0.031*
Hb (<12 g/dL)	1.97 (0.92-4.18)	0.081
LDL (>130 mg/dL)	1.46 (0.70-3.05)	0.308
HDL (<40 mg/dL)	2.22 (1.01-4.91)	0.048*
HbA1c (>8%)	1.29 (0.62-2.66)	0.488

## Discussion

The results of this investigation contributed to the body of knowledge showing a significant prevalence of vitamin B12 deficiency among T2DM patients receiving long-term metformin medication. Specifically, nearly one-third of participants (32.9%) were classified as vitamin B12-deficient. This deficiency demonstrated a clear association with both prolonged metformin treatment and higher daily dosages. These observations were consistent with global literature, which reports varying rates of metformin-associated vitamin B12 deficiency, ranging from approximately 20% to 66%, with differences often attributed to study population characteristics and the specific cutoff values used to define deficiency [14-17].

Metformin dosage and duration have both been shown to be independent predictors of vitamin B12 deficiency in several recent studies. This association was corroborated by the present multivariate logistic regression analysis, which demonstrated that metformin use for more than five years, higher daily doses, and concomitant pantoprazole use were significant risk factors. PPIs like pantoprazole can independently impair vitamin B12 absorption by reducing gastric acid secretion, and multiple studies have confirmed an additive or synergistic risk of deficiency when PPIs are combined with metformin [14,16,18-20].

The significance of taking into account combined drug effects on vitamin B12 absorption in patients with T2DM receiving long-term metformin therapy is highlighted by the independent association observed with concurrent pantoprazole use. Age, sex, glycemic control, and BMI did not significantly correlate with vitamin B12 deficiency in the current study. This finding aligned with several large-scale studies, in which these variables did not consistently predict vitamin B12 deficiency after adjusting for metformin exposure. Nevertheless, evidence from meta-analyses and cross-sectional studies indicates that all patients with T2DM on metformin remain at risk, regardless of demographic or clinical characteristics, highlighting the necessity for routine vitamin B12 monitoring [14-16,21].

Megaloblastic anemia is not the only clinical consequence of metformin-induced vitamin B12 deficiency. A lack of vitamin B12 may raise the risk of homocysteine-mediated cardiovascular disease, worsen or mimic diabetic peripheral neuropathy, and impair cognitive function. International guidelines like the American Diabetes Association Standards of Care 2024 [11] and the National Institute for Health and Care Excellence (NICE) 2024 guidelines [22], as well as recent narrative and systematic reviews, highlight the significance of routine vitamin B12 screening among metformin users, especially those taking higher doses or receiving treatment for more than four years.

The implications for clinical practice are therefore evident. Proactive identification and timely management of vitamin B12 deficiency may help prevent or attenuate neurological and hematological complications that are often attributed solely to diabetes, thereby improving patient quality of life. Despite increasing evidence and evolving guideline recommendations, vitamin B12 testing remains underutilized in diabetes care pathways globally, including in India. Early detection and cost-effective supplementation represent a practical and impactful preventive strategy, especially given the high prevalence of deficiency and its nonspecific clinical manifestations.

The use of defined laboratory cutoffs for vitamin B12 deficiency, a sufficient sample size, and multivariate logistic regression analysis to account for potential confounders are some of the study's strengths. The emphasis on the Indian population fills a significant information gap in the region because long-term metformin use and vitamin B12 insufficiency are both very common. Internal validity was further enhanced by strict inclusion and exclusion criteria, such as the removal of individuals taking vitamin B12 supplements or with disorders affecting vitamin B12 metabolism. 

There are a few limitations to be aware of. It is unable to establish causal or temporal correlations between metformin use and vitamin B12 insufficiency because of the cross-sectional design. Selection bias might exist in this single-center, hospital-based trial with a non-random sample, and the results might not apply to all individuals with T2DM. Without functional indicators like homocysteine or methylmalonic acid, serum vitamin B12 levels were employed, which would have resulted in an overestimate of subclinical deficiency. Furthermore, dietary parameters like vegetarianism were not evaluated, and medication history was partially reliant on patient reports, which could have led to residual confounding. Notwithstanding these drawbacks, the results offer clinically significant proof in favor of routine vitamin B12 testing for patients on long-term metformin therapy.

## Conclusions

Our study revealed that vitamin B12 deficiency was common among patients with T2DM and was independently associated with long-term metformin use and concomitant PPI therapy. Routine vitamin B12 screening in patients receiving prolonged or high-dose metformin may help prevent hematological and neurological complications and improve clinical outcomes.
